# Regulation of the Response of *Caenorhabditis elegans* to Simulated Microgravity by p38 Mitogen-Activated Protein Kinase Signaling

**DOI:** 10.1038/s41598-018-19377-z

**Published:** 2018-01-16

**Authors:** Wenjie Li, Daoyong Wang, Dayong Wang

**Affiliations:** Medical School, Southeast University, Nanjing, 210009 China

## Abstract

The *in vivo* function of p38 mitogen-activated protein kinase (MAPK) signaling in regulating the response to simulated microgravity is still largely unclear. Using *Caenorhabditis elegans* as an assay system, we investigated the *in vivo* function of p38 MAPK signaling in regulating the response of animals to simulated microgravity and the underlying molecular mechanism. Simulated microgravity treatment significantly increased the transcriptional expressions of genes (*pmk-1*, *sek-1*, and *nsy-1*) encoding core p38 MAPK signaling pathway and the expression of phosphorylated PMK-1/p38 MAPK. The *pmk-1*, *sek-1*, or *nsy-1* mutant was susceptible to adverse effects of simulated microgravity. The intestine-specific activity of PMK-1 was required for its function in regulating the response to simulated microgravity, and the entire p38 MAPK signaling pathway could act in the intestine to regulate the response to simulated microgravity. In the intestine, SKN-1 and ATF-7, two transcriptional factors, were identified as downstream targets for PMK-1 in regulating the response to simulated microgravity. Therefore, the activation of p38 MAPK signaling may mediate a protection mechanism for nematodes against the adverse effects of simulated microgravity. Additionally, our results highlight the potential crucial role of intestinal cells in response to simulated microgravity in nematodes.

## Introduction

Stress associated mitogen-activated protein kinase (MAPK) signaling cascades mainly contain p38 MAPK signaling, c-Jun N-terminal kinase (JNK) signaling, and ERK signaling based on kinase activity, target specificity, and protein homology. MAPK signaling can act as central signaling hubs by transducing extracellular cues and triggering specific cellular responses^1,2^. In organisms, MAPK signaling, such as the JNK signaling, regulates both normal and stress associated biological events^1,3,4^.

It has been well known that spaceflight will lead to the formation of significant risk for human beings and animals, such as alterations in movement, muscle atrophy, and metabolism^5–7^. Microgravity is one of the crucial contributors to these observed physiological changes^5,6^. *Caenorhabditis elegans* is a classic model animal for the study in the field of life sciences due to the properties of at least short life-cycle, short lifespan, and ease of culture^8^. Meanwhile, *C. elegans* is an ideal animal model for the study of physiological effects of simulated microgravity because of its common use on Earth as a model organism for human medical pathologies and its sensitivity to environmental toxicants or stresses^9–11^. In “the first International *C. elegans* Experiment in Space” (ICE-First) experiments, *C. elegans* has been employed to evaluate the potential different aspects of effects of spaceflight on animals^12–15^. It has been shown that simulated microgravity could affect early embryogenesis, reproduction, and locomotion behavior in nematodes^11–13,16–18^. Additionally, simulated microgravity may potentially cause the oxidative stress and DNA damage in nematodes^19,20^.

With the aid of different human cell lines, it was reported that expression of the p38 MAPK signaling could be significantly altered after simulated microgravity treatment^21–23^. Nevertheless, the *in vivo* function of p38 MAPK signaling in the regulation of response to simulated microgravity and the underlying mechanism are still largely unclear. In *C. elegans*, *pmk-1* encodes a MAPK, *sek-1* encodes a MAPK kinase (MAPKK), and *nsy-1* encodes a MAPK kinase kinase (MAPKKK), and these three proteins constitute the core p38 MAPK signaling pathway^2^. In nematodes, this p38 MAPK signaling is required for the control of pathogen response and stress response^24–27^. SKN-1/Nrf and ATF-7/bZIP usually act as downstream targets for PMK-1 in the regulation of different biological events^27–29^. Under normal conditions, the core p38 MAPK signaling does not affect longevity and locomotion behavior, and will not induce significant induction of reactive oxygen species (ROS) production in nematodes^27^. In the present study, we determined the *in vivo* function of p38 MAPK signaling pathway in regulating the response to simulated microgravity in nematodes using rotary wall vessel bioreactor in Synthecon Rotary System^TM^. Moreover, we examined the underlying molecular mechanism for p38 MAPK signaling in regulating the response of nematodes to simulated microgravity. Our results will be helpful for our understanding the *in vivo* function of p38 MAPK signaling in the regulation of response of organisms to simulated microgravity.

## Results

### Effect of simulated microgravity on expression of p38 MAPK signaling in wild-type nematodes

In this study, Synthecon Rotary System^TM^ was used as a simulated microgravity assay system. We set up two controls for simulated microgravity analysis, the control nematodes grown in liquid S medium and the control nematodes grown on normal nematode growth medium (NGM) plates. The control wild-type nematodes grown in liquid S medium showed the similar transcriptional expressions of genes (*nsy-1*, *sek-1*, and *pmk-1*) encoding p38 MAPK signaling pathway to those in control wild-type nematodes grown on normal NGM plates (Fig. S1a–c). In contrast, after simulated microgravity treatment, we observed the significant increase in transcriptional expressions of *nsy-1*, *sek-1*, and *pmk-1* in wild-type nematodes (Fig. S1a–c).

Since activation of p38 MAPK signaling usually requires the phosphorylation of p38 MAPK/PMK-1, we further compared the level of phosphorylated PMK-1 between control and simulated microgravity treated wild-type nematodes using Western blotting method. The control wild-type nematodes grown in liquid S medium had the similar expression of phosphorylated PMK-1 to that in control wild-type nematodes grown on normal NGM plates (Fig. S1d). In contrast, after simulated microgravity treatment, we observed a significant increase in the expression of phosphorylated PMK-1 in wild-type nematodes (Fig. S1d,e).

### Mutation of genes encoding p38 MAPK signaling pathway induced a susceptibility to simulated microgravity treatment

**Figure 1:**
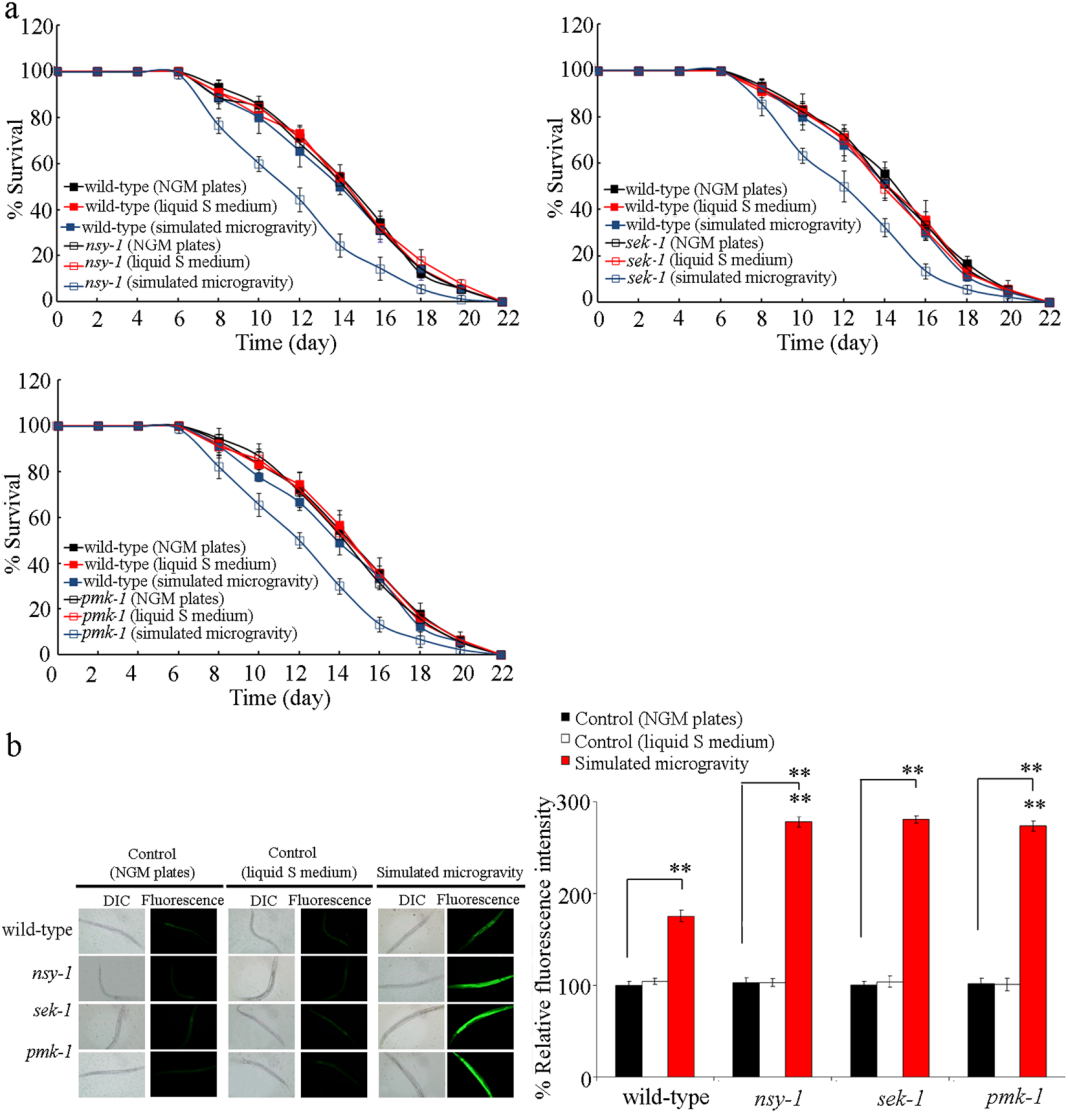
Mutation of genes encoding p38 MAPK signaling pathway induced a susceptibility to simulated microgravity treatment in nematodes. (**a**) Mutation of genes encoding p38 MAPK signaling pathway induced the reduced lifespan in simulated microgravity treated nematodes. (**b**) Mutation of genes encoding p38 MAPK signaling pathway induced a susceptibility to simulated microgravity treatment in inducing intestinal ROS production. Bars represent means ± SD. ^**^P < 0.01 *vs* wild-type (if not specially indicated).

We next employed the mutants for genes encoding the p38 MAPK signaling pathway to determine the function of p38 MAPK signaling pathway in regulating the response of nematodes to simulated microgravity. Intestinal ROS production and lifespan were selected as the toxicity assessment endpoints. In mammals, human cell lines, or nematodes, oxidative stress could be induced by simulated microgravity treatment^20,30,31^. Intestinal ROS production was used as an endpoint to reflect the induction of oxidative stress^32^. Lifespan was used to reflect the possible long-term effect of environmental toxicants or stresses^10,33^. In wild-type nematodes, simulated microgravity did not significantly affect the longevity (Figs 1a and S2, Table S1). The lifespan of wild-type nematodes grown in liquid S medium was similar to that on normal NGM plates under normal conditions (Figs 1a and S2, Table S1). Under normal conditions, mutation of *nsy-1*, *sek-1*, or *pmk-1* did not alter the longevity (Figs 1a and S2, Table S1). However, after the simulated microgravity treatment, mutation of *nsy-1*, *sek-1*, or *pmk-1* significantly reduced the lifespan (Figs 1a and S2, Table S1).

The wild-type nematodes grown in liquid S medium did not have the significant induction of intestinal ROS production as observed in wild-type nematodes grown on normal NGM plates under normal conditions (Fig. 1b). Similarly, the *nsy-1*, *sek-1*, and *pmk-1* mutants grown in liquid S medium or grown on normal NGM plates also did not have the significant induction of intestinal ROS production under normal conditions (Fig. 1b). In wild-type nematodes, simulated microgravity treatment could cause the significant induction of intestinal ROS production (Fig. 1b)^20^. Moreover, after simulated microgravity treatment, mutation of *nsy-1*, *sek-1*, or *pmk-1* induced the more severe induction of intestinal ROS production compared with that in wild-type nematodes (Fig. 1b).

In *C. elegans*, *gst-4* encoding a putative glutathione-requiring prostaglandin D synthase acting as a oxidative stress-response gene, which can be increased by paraquat, a ROS generator^34^. We further employed the transgenic strain of GST-4::GFP to examine the role of *nsy-1*, *sek-1*, or *pmk-1* in regulating the induction of ROS production in simulated microgravity treated nematodes. After simulated microgravity treatment, we observed a significant induction of GST-4::GFP expression (Fig. S3). In contrast, RNA interference (RNAi) knockdown of *pmk-1*, *sek-1*, or *nsy-1* dramatically decreased the induction of GST-4::GFP expression caused by simulated microgravity treatment (Fig. S3).

### Tissue-specific activity of PMK-1 in regulating the response of nematodes to simulated microgravity

**Figure 2:**
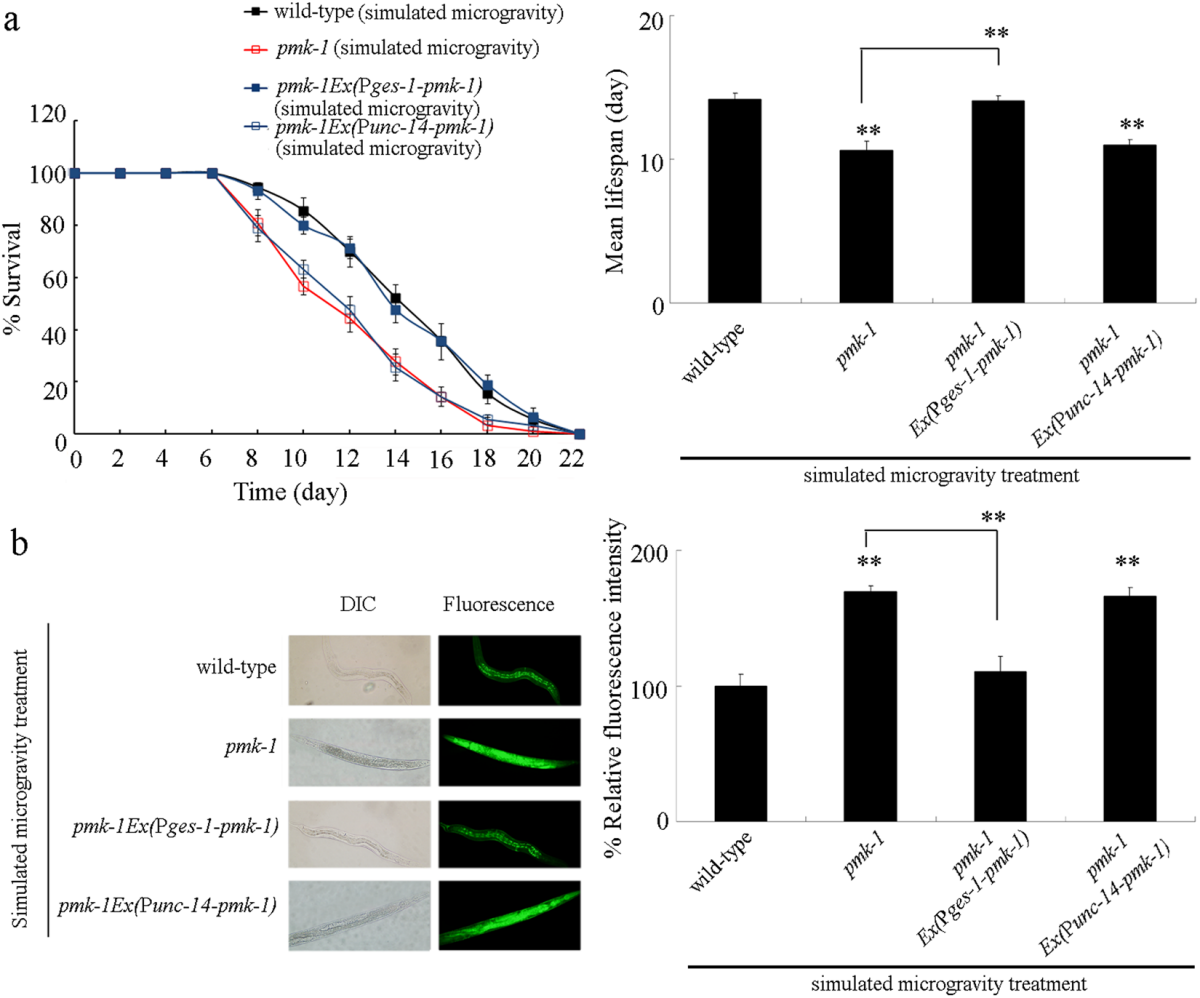
Tissue-specific activity of PMK-1 in regulating the response of nematodes to simulated microgravity. (**a**) Tissue-specific activity of PMK-1 in regulating lifespan in simulated microgravity treated nematodes. (**b**) Tissue-specific activity of PMK-1 in regulating the induction of intestinal ROS production in simulated microgravity treated nematodes. Bars represent means ± SD. ^**^P < 0.01 *vs* wild-type (if not specially indicated).

In *C. elegans*, *pmk-1* is broadly expressed in multiple tissues, including the intestine and the neurons^35^. We further determined the tissue-specific activity of PMK-1 in regulating the response of nematodes to simulated microgravity. Using lifespan and intestinal ROS production as the endpoints, we found that neuronal expression of *pmk-1* did not obviously affect the lifespan and the induction of intestinal ROS production in simulated microgravity treated *pmk-1* mutant nematodes (Fig. 2, Table S2). In contrast, we found that intestinal expression of *pmk-1* could significantly increase the lifespan and suppress the induction of intestinal ROS production in simulated microgravity treated *pmk-1* mutant nematodes (Fig. 2, Table S2).

### Effect of intestine-specific RNAi knockdown of genes encoding p38 MAPK signaling pathway on the response of nematodes to simulated microgravity

**Figure 3:**
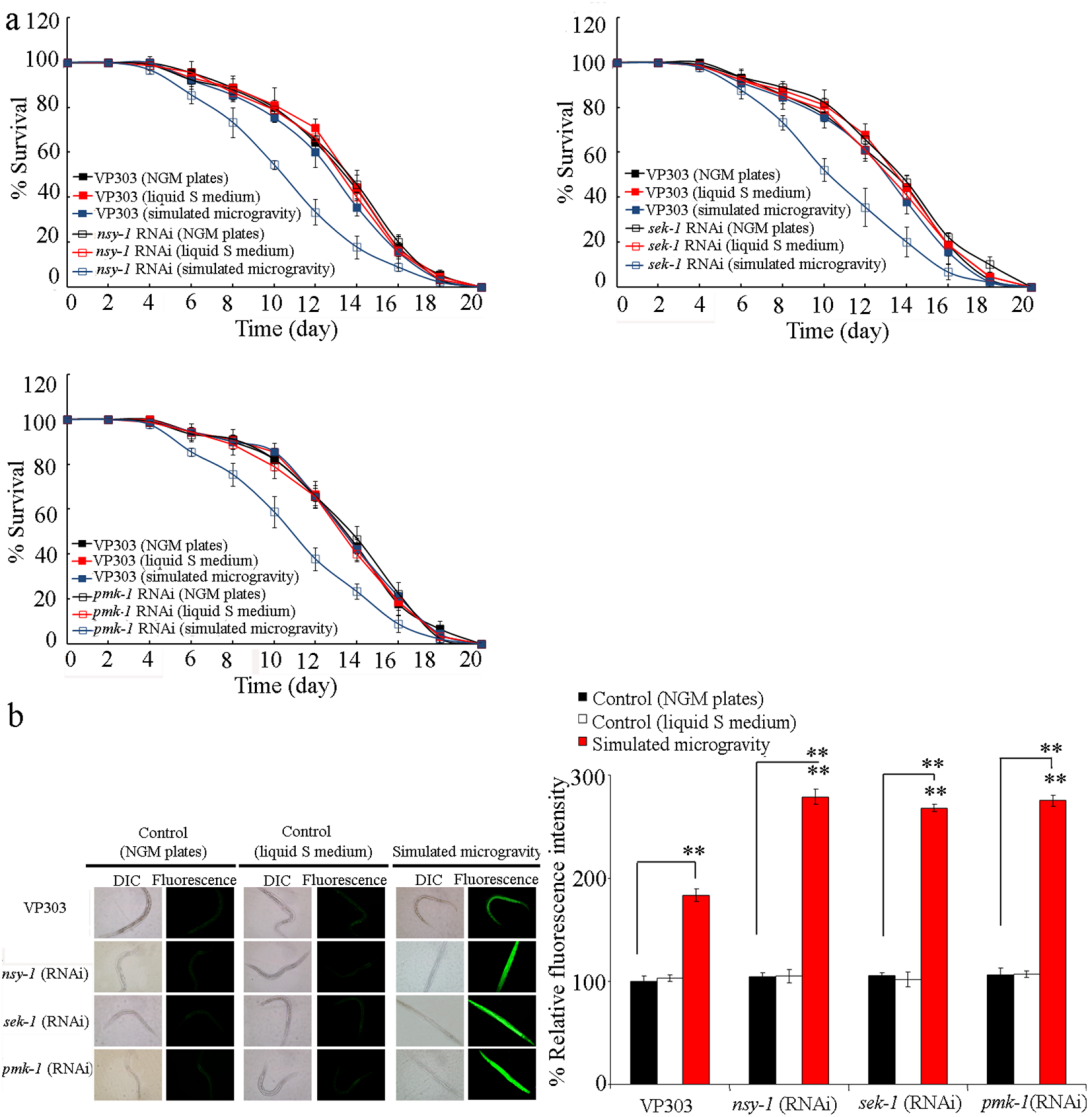
Effect of intestine-specific RNAi knockdown of genes encoding p38 MAPK signaling pathway on the response of nematodes to simulated microgravity. (**a**) Effect of intestine-specific RNAi knockdown of genes encoding p38 MAPK signaling pathway on the lifespan in simulated microgravity treated nematodes. (**b**) Effect of intestine-specific RNAi knockdown of genes encoding p38 MAPK signaling pathway on the induction of intestinal ROS production in simulated microgravity treatment. Bars represent means ± SD. ^**^P < 0.01 *vs* VP303 (if not specially indicated).

VP303 is an intestine-specific RNAi knockdown tool^36^. To confirm the intestinal function of genes encoding p38 MAPK signaling in the regulation of response to simulated microgravity, we further determined the effect of intestine-specific RNAi knockdown of genes encoding p38 MAPK signaling pathway on the response of nematodes to simulated microgravity. In VP303 strain, simulated microgravity did not significantly affect the longevity (Figs 3a and S4, Table S3). The lifespan of VP303 strain grown in liquid S medium was similar to that on normal NGM plates under normal conditions (Figs 3a and S4, Table S3). Both VP303 strain grown in liquid S medium and VP303 strain grown on normal NGM plates did not show the significant induction of intestinal ROS production under normal conditions (Fig. 3b). Under normal conditions, RNAi knockdown of *nsy-1*, *sek-1*, or *pmk-1* did not affect the longevity (Figs 3a and S4, Table S3). However, after simulated microgravity treatment, RNAi knockdown of *nsy-1*, *sek-1*, or *pmk-1* significantly reduced the lifespan (Fig. 3a, Table S3). Under normal conditions, RNAi knockdown of *nsy-1*, *sek-1*, or *pmk-1* did not cause the significant induction of intestinal ROS production (Fig. 3b). In VP303 strain, simulated microgravity treatment resulted in the significant induction of intestinal ROS production (Fig. 3b). In contrast, after simulated microgravity treatment, RNAi knockdown of *nsy-1*, *sek-1*, or *pmk-1* led to the more severe induction of intestinal ROS production compared with that in VP303 strain (Fig. 3b).

### Intestinal overexpression of PMK-1 induced a resistance to simulated microgravity treatment

**Figure 4:**
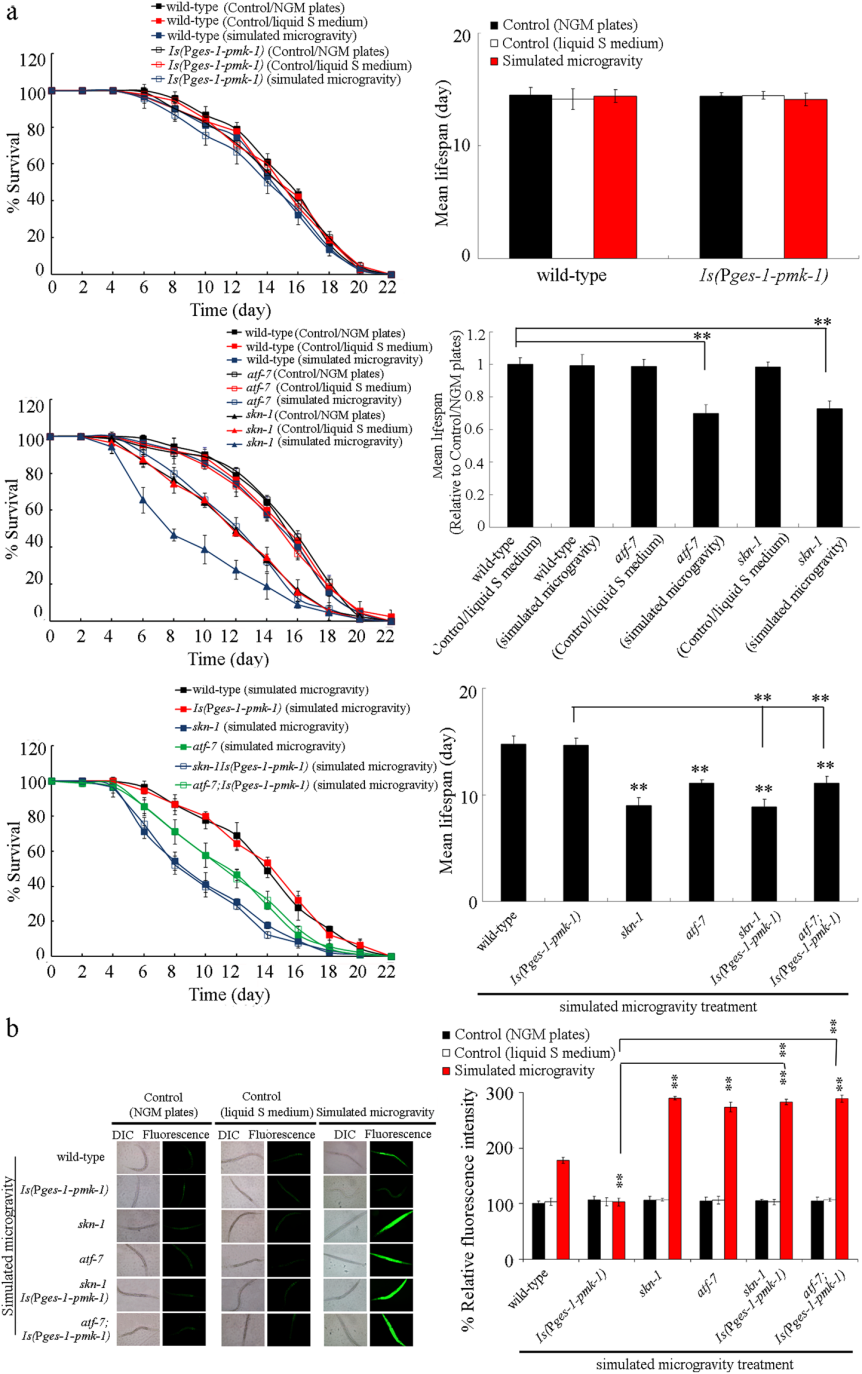
Genetic interaction between PMK-1 and SKN-1 or ATF-7 in regulating the response of nematodes to simulated microgravity. (**a**) Genetic interaction between PMK-1 and SKN-1 or ATF-7 in regulating the lifespan in simulated microgravity treated nematodes. (**b**) Genetic interaction between PMK-1 and SKN-1 or ATF-7 in regulating the induction of intestinal ROS production in simulated microgravity treatment. Bars represent means ± SD. ^**^P < 0.01 *vs* wild-type (if not specially indicated).

We also investigated the effect of intestinal overexpression of PMK-1 on the response of nematodes to simulated microgravity. Under normal conditions, the nematodes overexpressing intestinal PMK-1 exhibited the similar lifespan to that in wild-type nematodes, and could not cause the significant induction of intestinal ROS production (Fig. 4, Table S4). After simulated microgravity treatment, although intestinal overexpression of PMK-1 did not obviously alter the lifespan, intestinal overexpression of PMK-1 significantly suppressed the induction of intestinal ROS production observed in simulated microgravity treated wild-type nematodes (Fig. 4, Table S4). Therefore, intestinal overexpression of PMK-1 can potentially induce a resistance to simulated microgravity treatment.

### Mutation of *skn-1* or *atf-7* induced a susceptibility to simulated microgravity treatment

The wild-type nematodes grown in liquid S medium showed the similar transcriptional expressions of *skn-1* and *atf-7*, two potential targeted genes of *pmk-1*^27–29^, to those in control wild-type nematodes grown on normal NGM plates (Fig. S5a–c). After simulated microgravity treatment, we detected the significant increase in transcriptional expressions of *skn-1* and *atf-7* in wild-type nematodes (Fig. S5a–c). Meanwhile, we observed the obvious translocation of SKN-1::GFP into the nucleus after simulated microgravity treatment (Fig. S5d). In organisms, Nrf proteins have the cellular protective function by acting as a regulator of antioxidant or xenbiotic defense^37^. ATF-7, a bZIP transcription factor, could act downstream of PMK-1 to regulate innate immunity^29^.

After simulated microgravity treatment, we detected the significant decrease in relative mean lifespan (treatment/Control(NGM plates)) in *skn-1* or *atf-7* mutant nematodes compared with that in wild-type nematodes (Fig. 4a, Table S4). Under normal conditions, mutation of *skn-1* or *atf-7* did not result in the significant induction of intestinal ROS production (Fig. 4b). After simulated microgravity treatment, we observed more severe induction of intestinal ROS production in *skn-1* or *atf-7* mutant nematodes compared with that in wild-type nematodes (Fig. 4b). Moreover, we found that RNAi knockdown of *skn-1* or *atf-7* also dramatically suppressed the induction of GST-4::GFP expression caused by simulated microgravity treatment (Fig. S3). These results suggest that mutation of *skn-1* or *atf-7* may induce a susceptibility to simulated microgravity treatment.

### Genetic interaction between PMK-1 and SKN-1 or ATF-7 in regulating the response to simulated microgravity

To determine whether SKN-1 and ATF-7 act downstream of PMK-1 in the regulation of response to simulated microgravity, we investigated the genetic interaction between PMK-1 and SKN-1 or ATF-7 in regulating the response to simulated microgravity. After simulated microgravity treatment, we found that *skn-1* or *atf-7* mutation could significantly reduce the lifespan and increase the induction of intestinal ROS production in transgenic strain overexpressing intestinal *pmk-1* (Fig. 4, Table S4).

### Effect of intestine-specific RNAi knockdown of *skn-1* or *atf-7* on the response of nematodes to simulated microgravity

**Figure 5:**
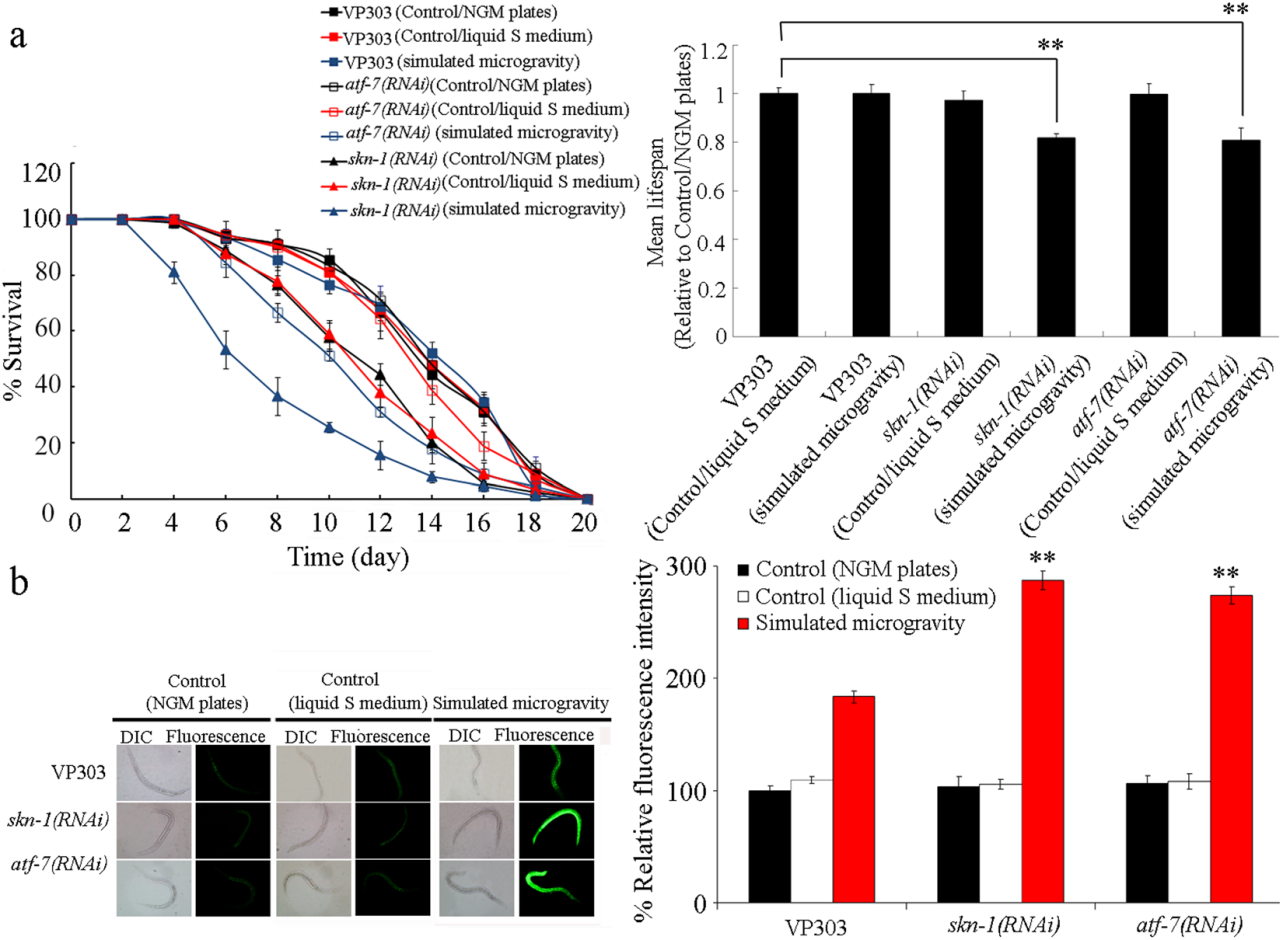
Effect of intestine-specific RNAi knockdown of *skn-1* or *atf-7* on the response of nematodes to simulated microgravity. (**a**) Effect of intestine-specific RNAi knockdown of *skn-1* or *atf-7* on the lifespan in simulated microgravity treated nematodes. (**b**) Effect of intestine-specific RNAi knockdown of *skn-1* or *atf-7* on the induction of intestinal ROS production in simulated microgravity treatment. Bars represent means ± SD. ^**^P < 0.01 *vs* VP303 (if not specially indicated).

Similarly, after simulated microgravity treatment, we found the significant decrease in relative mean lifespan (treatment/Control(NGM plates)) in nematodes with intestine-specific RNAi knockdown of *skn-1* or *atf-7* compared with that in VP303 strain (Fig. 5a, Table S5). Under normal conditions, intestine-specific RNAi knockdown of *skn-1* or *atf-7* could not induce the significant intestinal ROS production (Fig. 5b). After simulated microgravity treatment, we observed the more severe induction of intestinal ROS production in nematodes with intestine-specific RNAi knockdown of *skn-1* or *atf-7* compared with that in VP303 strain (Fig. 5b). Therefore, intestine-specific RNAi knockdown of *skn-1* or *atf-7* also induce a susceptibility to simulated microgravity treatment.

## Discussion

In this study, using *C. elegans* as the assay system, we first investigated the *in vivo* role of p38 MAPK signaling in response to simulated microgravity. In nematodes, we observed the significant increase in transcriptional expressions of genes (*pmk-1*, *sek-1*, and *nsy-1*) encoding the core p38 MAPK signaling pathway after simulated microgravity treatment (Fig. S1a–c). This observation is largely consistent with the *in vitro* data on the response of p38 MAPK signaling to simulated microgravity treatment in different human cell lines^21–23^. Therefore, both *in vitro* and *in vivo* evidence have suggested the response of p38 MAPK signaling to simulated microgravity with the increased expression tendency.

Considering the great value of *C. elegans* as a model animal, we here further determined the *in vivo* function of p38 MAPK signaling in the regulation of response of animals to simulated microgravity. In nematodes, with the aid of corresponding loss-of-function mutants, we found that mutation of *pmk-1*, *sek-1*, or *nsy-1* resulted in the formation of a susceptibility to simulated microgravity treatment using lifespan and intestinal ROS production as the endpoints (Figs 1 and S2). Therefore, our data suggest the involvement of core p38 MAPK signaling cascade (NSY-1-SEK-1-PMK-1) in the activation of protective response of nematodes to simulated microgravity treatment. In *C. elegans*, simulated microgravity treatment could significantly decrease the transcriptional expressions of *mev-1* and *gas-1* (Fig. S6). MEV-1, an ortholog of succinate dehydrogenase cytochrome b560 subunit of mitochondrial respiratory chain complex II, and GAS-1, a subunit of mitochondrial complex I, are two protein components in mitochondrial complex required for the function of primary molecular machinery in regulating the oxidative stress^38,39^. Therefore, simulated microgravity treatment may at least cause the adverse effects on the mitochondrial functions, which in turns induces the significant ROS production in nematodes. Meanwhile, we observed that simulated microgravity treatment could significantly increase the transcriptional expressions of *sod-2*, *sod-3*, *sod-4*, and *sod-5* genes (Fig. S6). Nevertheless, our data imply that the observed increase in *sod* genes may be not enough to counteract the adverse effects induced by simulated microgravity treatment. In *C. elegans*, *sod* genes encode superoxide dismutases (SODs) including the mitochondrial SODs provide the antioxidative defense system for nematodes against the oxidative stress^40^.

In this study, we further determined the tissue-specific activity of PMK-1/p38 MAPK in the regulation of response to simulated microgravity. Our data suggest that the activity of PMK-1 in the intestine was required for the regulation of response to simulated microgravity (Fig. 2). In contrast, the neuronal activity of PMK-1 was not necessary during the regulation of response to simulated microgravity (Fig. 2). Based on the data on intestinal activity of PMK-1, our results imply that the intestinal cells may play a key role in response to simulated microgravity in nematodes.

Using VP303 strain as an intestine-specific RNAi tool, we further found that intestine-specific RNAi knockdown of *pmk-1*, *sek-1*, or *nsy-1* induced a susceptibility to simulated microgravity treatment (Figs 3 and S4). These results imply that simulated microgravity may activate the response of entire p38 MAPK signaling pathway in the intestine of nematodes. That is, the entire p38 MAPK signaling pathway in the intestine was involved in the regulation of response to simulated microgravity.

**Figure 6:**
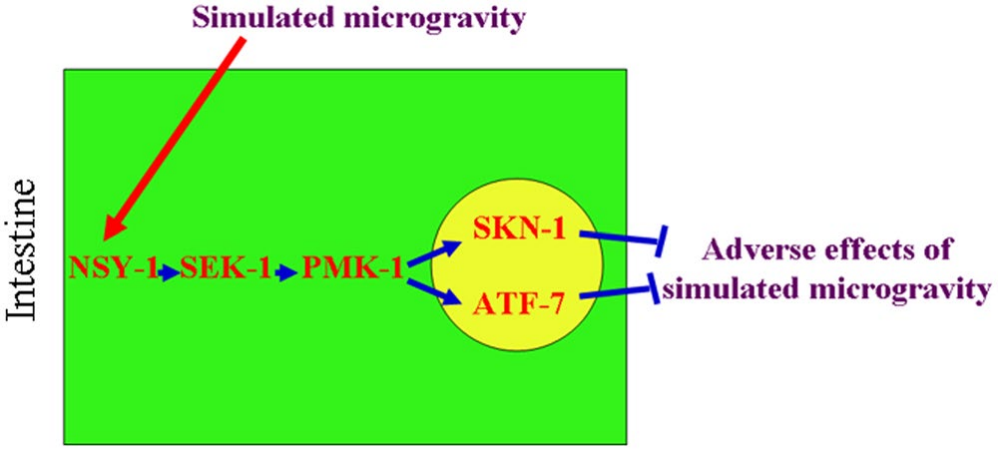
A diagram showing the role of signaling cascade of NSY-1-SEK-1-PMK-1-SKN-1/ATF-7 in the regulation of response to simulated microgravity.

In this study, we further provide the underlying molecular mechanism for p38 MAPK signaling in the regulation of response to simulated microgravity. We found that mutation of *skn-1* encoding a Nrf transcription factor or *atf-7* encoding a bZIP transcription factor could significantly suppress the resistance of transgenic strain overexpressing intestinal *pmk-1* to simulated microgravity treatment (Fig. 4), which suggest that intestinal PMK-1 may regulate the response of nematodes to simulated microgravity by affecting the activity of SKN-1 or ATF-7 (Fig. 6). Some other results further support this. We observed the significant increase in transcriptional expressions of *skn-1* and *atf-7* in simulated microgravity treated wild-type nematodes (Fig. S5). Additionally, like the effects from mutation or intestine-specific RNAi knockdown of *pmk-1*, *sek-1*, or *nsy-1*, mutation or intestine-specific RNAi knockdown of *skn-1* or *atf-7* also induced a susceptibility to simulated microgravity treatment (Figs 4 and 5). Therefore, we raised the signaling cascade of NSY-1-SEK-1-PMK-1-SKN-1/ATF-7 required for the regulation of response to simulated microgravity (Fig. 6).

In conclusion, we here investigated the *in vivo* function of p38 MAPK signaling in the regulation of response to simulated microgravity and the underlying molecular mechanism using the *C. elegans* assay system. In nematodes, simulated microgravity could significantly increase the expression of p38 MAPK signaling. Meanwhile, mutation of *pmk-1*, *sek-1*, or *nsy-1* in the p38 MAPK signaling pathway caused the susceptibility to simulated microgravity treatment. Therefore, the activation of p38 MAPK signaling may mediate a protection mechanism for animals against the adverse effects from simulated microgravity treatment. We further provided the evidence to demonstrate the involvement of intestine-specific activity of p38 MAPK signaling in the regulation of response to simulated microgravity. In the intestine, the signaling cascade of NSY-1-SEK-1-PMK-1-SKN-1/ATF-7 was raised to be required for the regulation of response to simulated microgravity. Our results highlight the potential key role of intestinal cells in response to simulated microgravity in animals.

## Methods

### *C. elegans* strains and maintenance

*C. elegans* strains used in this study were wild-type N2, mutants of *skn-1*(*zj15*), *atf-7*(*tm4392*), *pmk-1*(*km25*), *sek-1*(*km4*), and *nsy-1*(*ag3*), and transgenic strains of *Is*(P*ges-1-pmk-1*)^27^, *pmk-1*(*km25*)*Ex*(P*ges-1-pmk-1*), *pmk-1*(*km25*)*Ex*(*Punc-14-pmk-1*), *skn-1*(*zj15*)*Is*(P*ges-1-pmk-1*), *atf-7*(*tm4392*)*;Is*(P*ges-1-pmk-1*), CL2166/*dvIs19*[gst-4::GFP], LD1/*idIs7*[skn-1::GFP], and VP303/*kbIs7*[*nhx-2p::rde-1*]. Some of the strains were obtained from *Caenorhabditis* Genetics Center (funded by NIH Office of Research Infrastructure Programs (P40 OD010440)). The mutants were out-crossed for at least four times. Nematode strains were maintained on normal NGM plates seeded with *Escherichia coli* OP50 (a food source) at 20 °C as described^8^. Gravid nematodes were lysed with a bleaching mixture (0.45 M NaOH, 2% HOCl) in order to separate the eggs and the animals. The collected eggs were allowed to develop into age synchronous L1-larvae or young adult populations.

### Simulated microgravity treatment

After the transfer of approximately 100 young adults into liquid S medium in the presence of OP50 in the cultivation chamber in the Rotary System^TM^ developed by National Aeronautics and Space Administration (Synthecon, Houston, TX, USA), the vessels were half filled. Simulated microgravity was generated by suspending nematodes in S medium in this assay system after balancing their sedimentation-induced gravity with centrifugation caused by Rotary Cell Culture System (RCCS) vessel rotation (30 rpm)^41^. The RCCS will rotate the culture chamber horizontally. The simulated microgravity treatment was performed for 24 h at 20 °C. The control nematodes were grown in liquid S medium in the presence of OP50 under the condition of normal gravitational force (1 G) or on normal NGM plates at 20 °C. In this assay system, the simulated microgravity-treated young adults have the reproductive capacity (data not shown).

### Toxicity assessment

ROS production was analyzed as described previously^42,43^. The examined nematodes were incubated with 1 μM 5′,6′-chloromethyl-2′,7′-dichlorodihydro-fluorescein diacetate (CM-H_2_DCFDA; Molecular Probes) solution for 3 h at 20 °C in the dark. After the labeling, the examined nematodes were washed with M9 buffer for three times. And then, the nematodes were mounted on a 2% agar pad, and analyzed at 488 nm of excitation wavelength and at 510 nm of emission filter under a laser scanning confocal microscope (Leica, TCS SP2, Bensheim, Germany). Relative fluorescence intensity of ROS signals was semi-quantified and expressed as the value relative to total protein concentration^44^. Ten independent trails were performed, and thirty nematodes were examined for each trail.

The lifespan was analyzed at 20 °C as described previously^45,46^. After simulated microgravity treatment, the nematodes were transferred to normal NGM plates. After that, we started to record the lifespan data. During the lifespan assay, hermaphrodites were transferred daily for the first 7 d of adulthood. The examined nematodes were checked every two-day. The examined nematodes would be scored as dead, if they did not move even after repeated taps with a pick. Fifty nematodes were examined per treatment for the survival percent assay, and three replicates were performed. Survival curve data were statistically analyzed using the log-rank test.

### Reverse-transcription and quantitative real-time polymerase chain reaction (qRT-PCR) assay

Total RNA of nematodes was isolated using the reagent of Trizol (Invitrogen, UK) according to the manufacturer’s protocol. Purity and concentration of the isolated RNAs were evaluated by a ratio of OD260/280 using a spectrophotometer. After the cDNA synthesis, transcriptional expressions of the examined genes were determined by real-time PCR in an ABI 7500 real-time PCR system with Evagreen (Biotium, USA). Relative quantification of targeted genes was expressed as transcriptional expression ratio between the examined genes and the reference gene of *tba-1* encoding alpha-tubulin protein, *pmp-3* encoding a putative ABC transporter, or *act-1* encoding an actin. All the reactions were performed in triplicate, and the replicates are biological. Primer information for real-time PCR of the examined genes and the reference gene was shown in Table S6.

### Western blotting assay

Protein extracted from nematodes was electrophoresed on a 10% sodium dodecyl sulfate-polyacrylamide gel electrophoresis (SDS-PAGE) gel. After the electrophoresis, the gel was transferred to a nitrocellulose membrane in a Bio-Rad (Hercules, CA) semi-dry transfer apparatus. The membrane was pre-incubated with 5% nonfat milk in TBST buffer (10 mM Tris, pH 8.0, 150 mM NaCl and 0.5% Tween 20) for 30 min at room temperature. After that, the membrane was incubated with primary antibody (Anti-phospho-p38 MAPK monoclonal antibody (1:500, Cell Signaling), or anti-Actin monoclonal antibody (1:5000, EMD Millipore)) in TBST buffer with 5% nonfat milk for 12 h at 4 °C. After washing with TBST buffer for three times (10 min each time), the membrane was incubated with horseradish peroxidase (HRP)-conjugated secondary antibody (goat anti-mouse IgG antibody (H&L) [HRP] (1:10 000, GenScript)) for 1.5 h at room temperature. The membrane was developed with ECL system (Thermo Scientific, Pittsburgh, PA). Three replicates were performed.

### RNAi

RNAi was performed by feeding nematodes with *E. coli* strain HT115 (DE3) expressing double-stranded RNA that is homologous to a certain gene as described^47^. HT115 (DE3) grown in LB broth containing ampicillin (100 mg/mL) was plated onto NGM plates containing ampicillin (100 mg/mL) and isopropyl 1-thio-b-D-galactopyranoside (IPTG, 5 mM). L1 larvae were placed on RNAi plates until the nematodes became gravid, which were transferred to fresh RNAi-expressing bacterial lawns to lay eggs so as to obtain the second generation of RNAi population. Eggs were then allowed to develop at 20 °C to young adults for the subsequent assays. The second generation was cultured onto non-RNAi plates. Animals grown on HT115 bacteria expressing the empty vector L4440 was used as a control.

### DNA constructs and transformation

To generate entry vector carrying promoter sequence, promoter region for *unc-14* gene specially expressed in the neurons or *ges-1* gene specially expressed in the intestine was amplified by PCR from wild-type *C. elegans* genomic DNA. The promoter fragment was inserted into the pPD95_77 vector in the sense orientation, and *pmk-1* cDNA was inserted into corresponding entry vector after the promoter sequence. Germline transformation was performed by coinjecting a testing DNA (10–40 μg/mL) and a marker DNA of P*dop-1::rfp* (60 μg/mL) into the gonad of nematodes as described^48^. The related primer information for DNA constructions were shown in Table S7.

### Statistical analysis

All data in this article were expressed as means ± standard deviation (SD). Graphs were generated using Microsoft Excel (Microsoft Corp., Redmond, WA). Statistical analysis was performed using SPSS 12.0 (SPSS Inc., Chicago, USA). Differences between groups were determined using one-way analysis of variance (ANOVA). Probability levels of 0.05 (^*^) and 0.01 (^**^) were considered to be statistically significant.

## Electronic supplementary material

Supporting information
