# Supplementary material for: Regulation of the Response of Caenorhabditis elegans to Simulated Microgravity by p38 Mitogen-Activated Protein Kinase Signaling
Source: Sci Rep. 2018 Jan 16;8:857. doi: 10.1038/s41598-018-19377-z (PMC5770453; doi:10.1038/s41598-018-19377-z)
Supplement: Supplementary file 1 — Supporting information [file 41598_2018_19377_MOESM1_ESM.doc]

**Regulation of the Response of *Caenorhabditis elegans* to Simulated Microgravity by p38 Mitogen-Activated Protein Kinase Signaling**

Wenjie Li, Daoyong Wang & Dayong Wang^*^

Medical School, Southeast University, Nanjing 210009, China

^*^Correspondence and requests for materials should be addressed to D.W. (email: [dayongw@seu.edu.cn](mailto:dayongw@seu.edu.cn)).

**Supporting Information:**

**
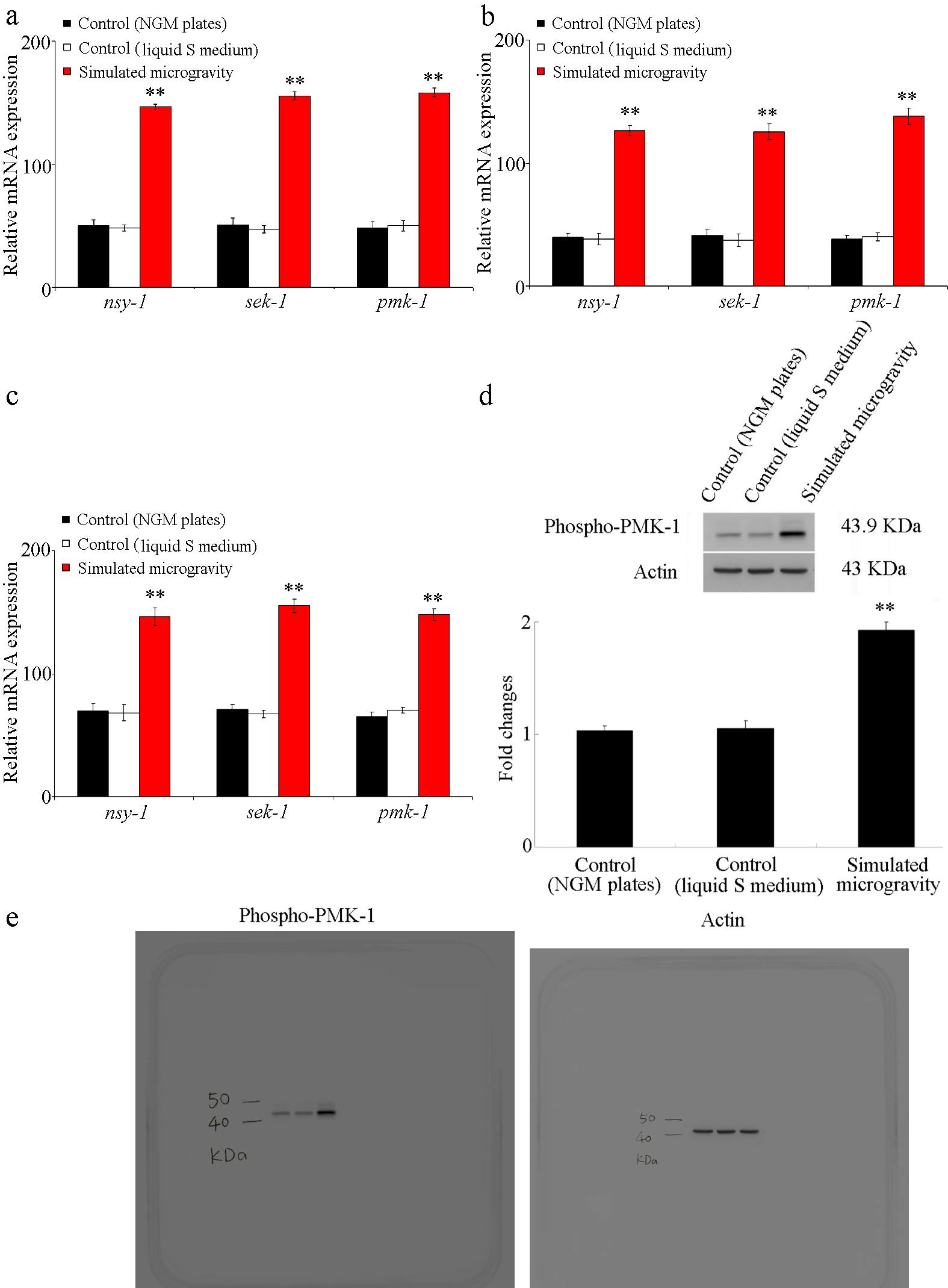
**

**Figure S1.** **Effect of simulated microgravity on expression of p38 MAPK signaling in wild-type nematodes.**  (**a**) Effect of simulated microgravity on expression of p38 MAPK signaling in wild-type nematodes using *tba-1* as a reference gene. (**b**) Effect of simulated microgravity on expression of p38 MAPK signaling in wild-type nematodes using *pmp-3* as a reference gene. (**c**) Effect of simulated microgravity on expression of p38 MAPK signaling in wild-type nematodes using *act-1* as a reference gene. (**d**) Western blotting analysis of the effect of simulated microgravity on expression level of phosphorylated PMK-1. (**e**) The original gel images for Figure S1d. Bars represent means ± SD. ^**^*P* < 0.01 *vs* control (NGM plates).

**
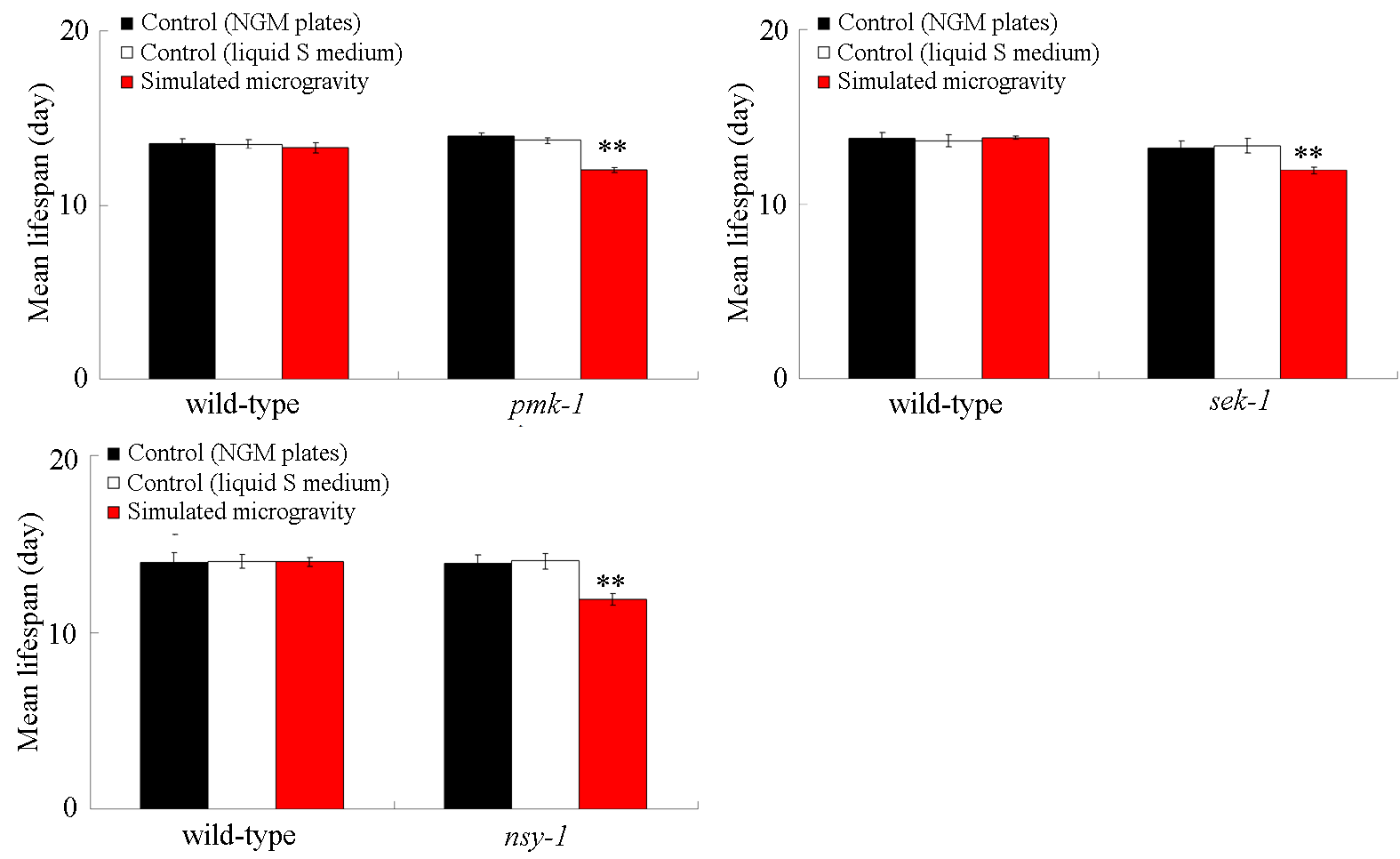
**

**Figure S2. Mutation of genes encoding p38 MAPK signaling pathway reduced the lifespan in simulated microgravity treated nematodes.** Bars represent means ± SD. ^**^*P* < 0.01 *vs* wild-type.


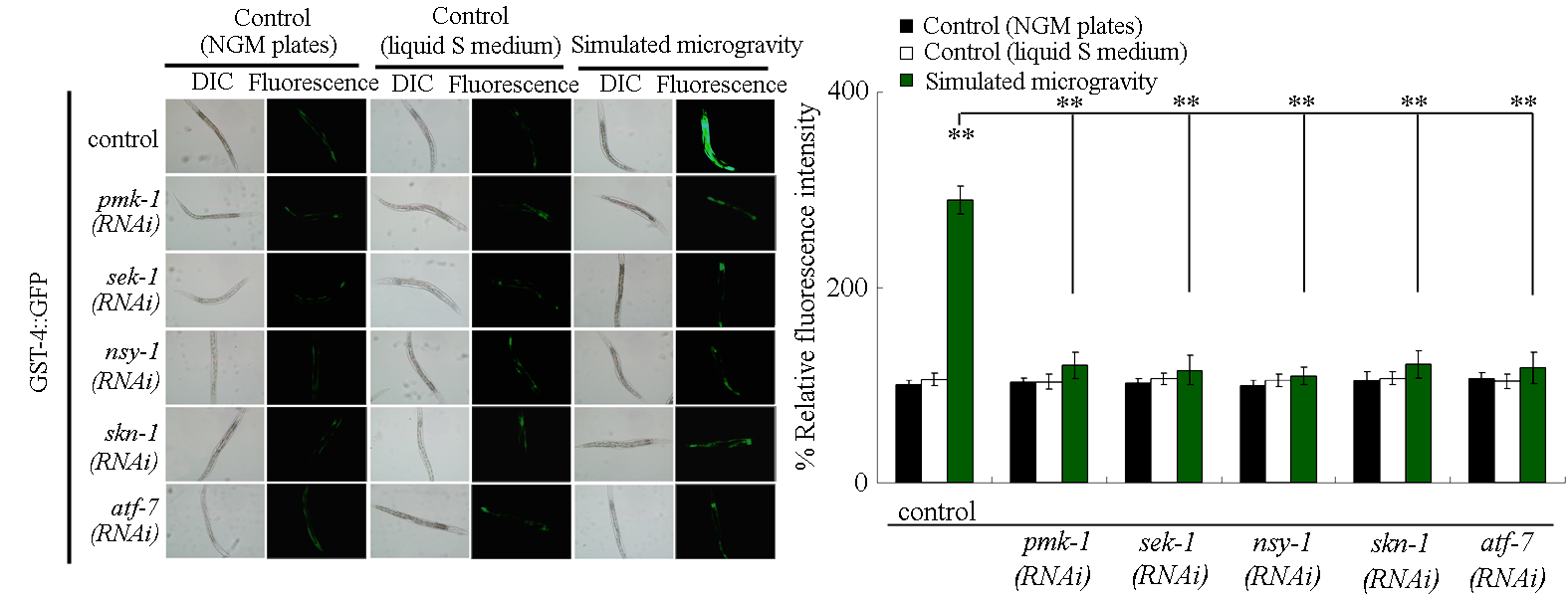


**Figure S3 Comparison of GST-4::GFP expression.** Bars represent means ± SD. ^**^*P* < 0.01 *vs* Control (NGM plates) (if not specially indicated).

**
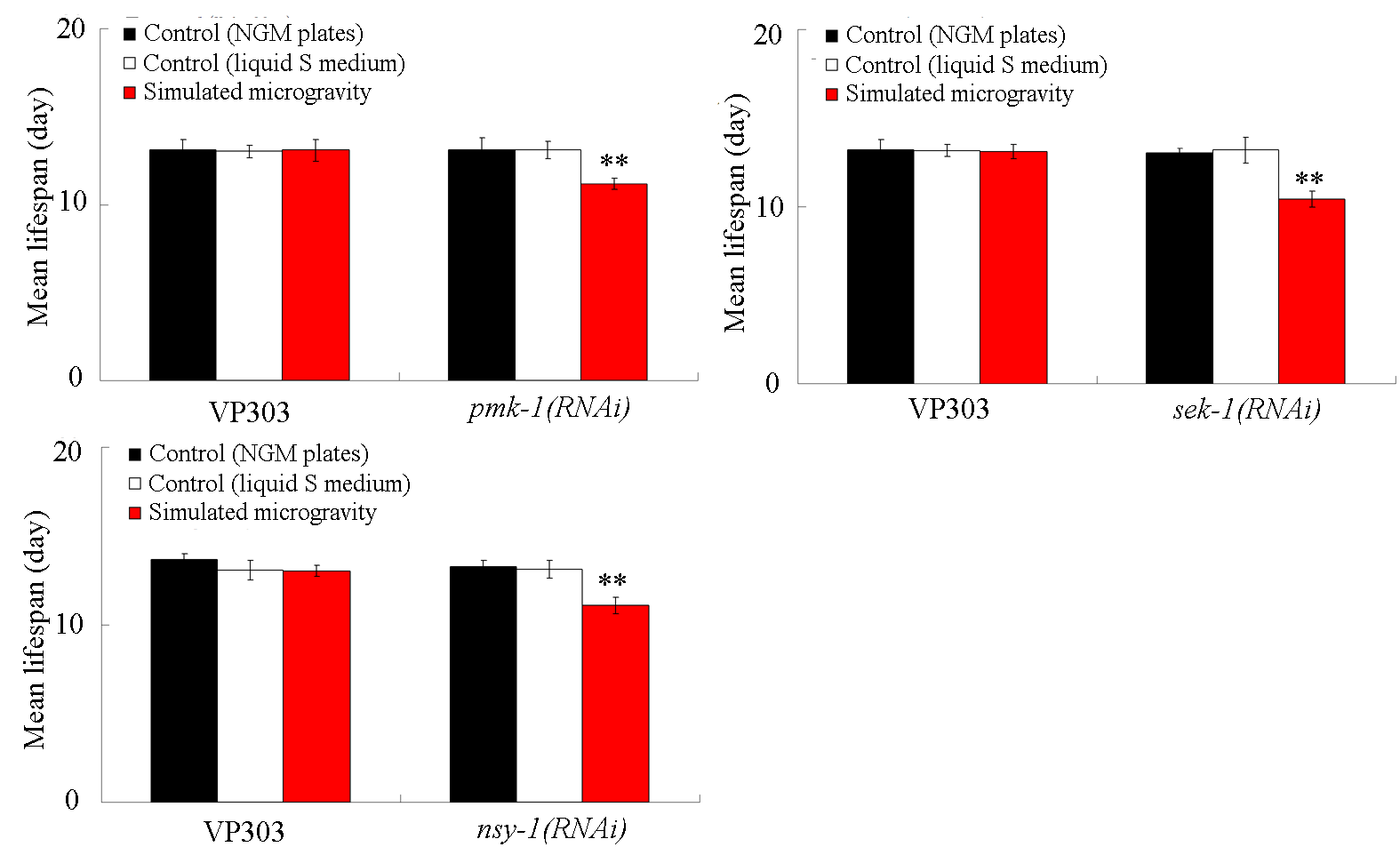
**

**Figure S4. Effect of intestine-specific RNAi knockdown of genes encoding p38 MAPK signaling pathway on the lifespan in simulated microgravity treated nematodes.**  Bars represent means ± SD. ^**^*P* < 0.01 *vs* VP303.

**
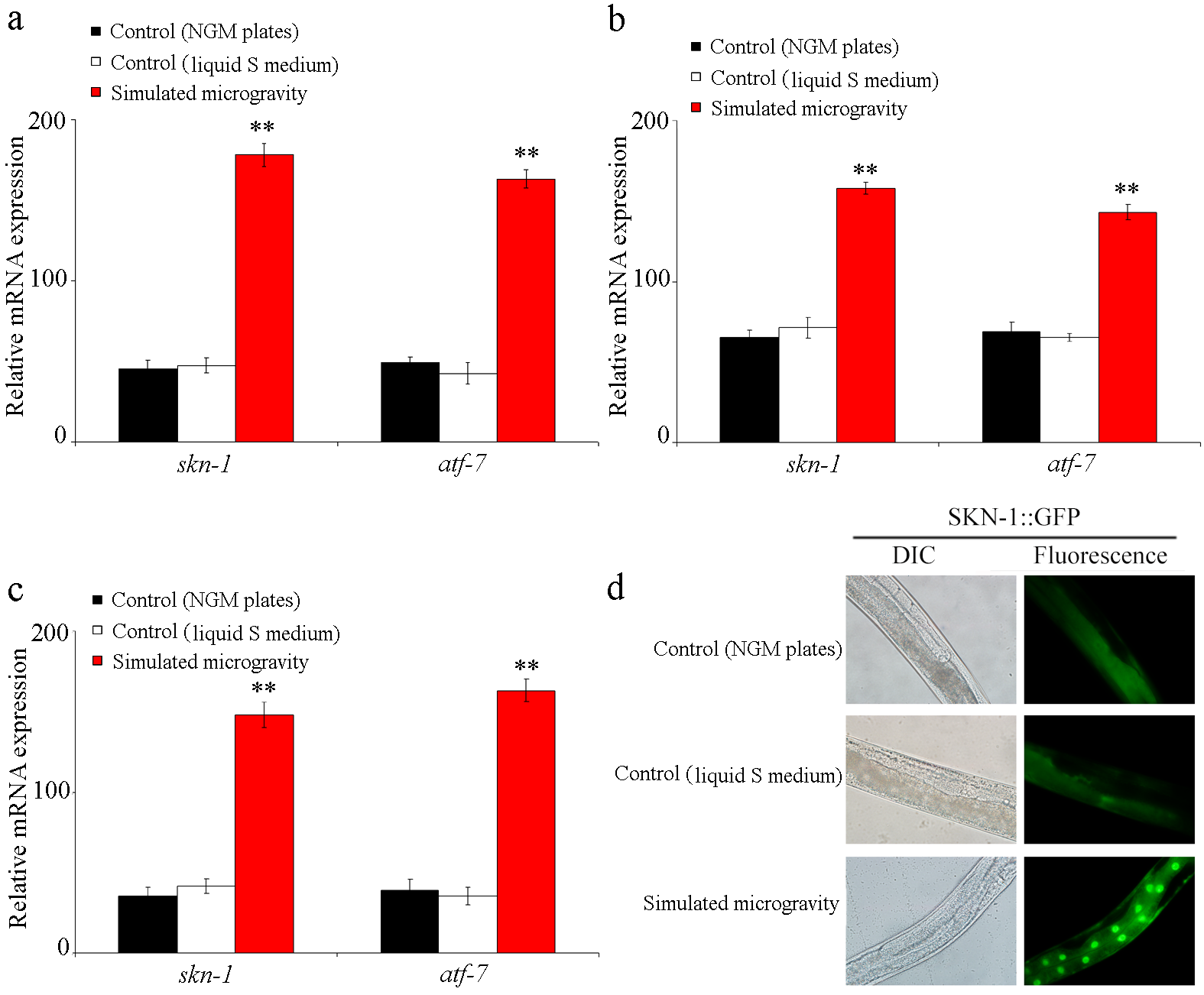
**

**Figure S5. Effect of simulated microgravity on transcriptional expressions of *skn-1* and *atf-7*.** (**a**) Effect of simulated microgravity on expression of *skn-1* and *atf-7* in wild-type nematodes using *tba-1* as a reference gene. (**b**) Effect of simulated microgravity on expression of *skn-1* and *atf-7* in wild-type nematodes using *pmp-3* as a reference gene. (**c**) Effect of simulated microgravity on expression of *skn-1* and *atf-7* in wild-type nematodes using *act-1* as a reference gene. (**d**) Effect of simulated microgravity on SKN-1::GFP expression. Bars represent means ± SD. ^**^*P* < 0.01 *vs* control (NGM plates).


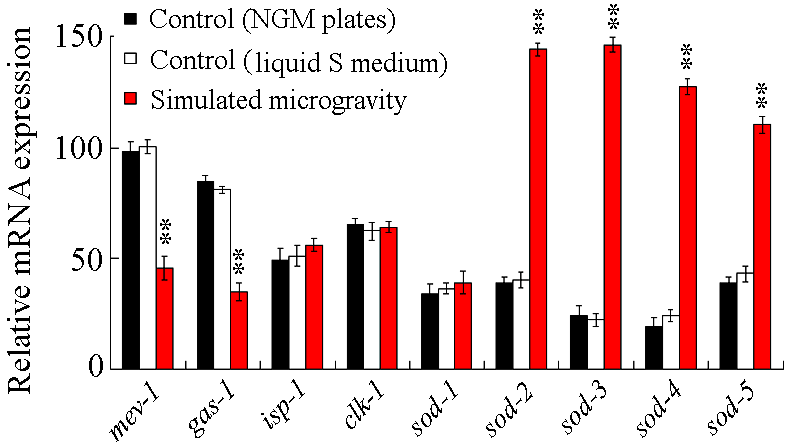


**Figure S6 Effect of simulated microgravity on expression of *mev-1*, *gas-1*, *isp-1*, *clk-1*, and *sod* genes in wild-type nematodes using *tba-1* as a reference gene.** Bars represent means ± SD. ^**^*P* < 0.01 *vs* control (NGM plates).

**Table S1. Mutation of genes encoding p38 MAPK signaling pathway reduced the lifespan in simulated microgravity treated nematodes**

|  | Mean lifespan (day) | Significance (*vs.* Control/NGM plates) | Significance (*vs.* wild-type) |
| --- | --- | --- | --- |
| wild-type (Control/NGM plates) | 13.54 ± 0.3 |  |  |
| wild-type (liquid S medium) | 13.5 ± 0.252 | NS |  |
| wild-type (simulated microgravity) | 13.28 ± 0.304 | NS |  |
| *pmk-1* (Control/NGM plates) | 13.97 ± 0.2 |  | NS |
| *pmk-1* (liquid S medium) | 13.7 ± 0.163 | NS | NS |
| *pmk-1* (simulated microgravity) | 11.99 ± 0.134 | *P* < 0.01 | *P* < 0.01 |
|  |  |  |  |
| wild-type (Control/NGM plates) | 13.75 ± 0.44 |  |  |
| wild-type (liquid S medium) | 13.6 ± 0.352 | NS |  |
| wild-type (simulated microgravity) | 13.78 ± 0.104 | NS |  |
| *sek-1* (Control/NGM plates) | 13.2 ± 0.344 |  | NS |
| *sek-1* (liquid S medium) | 13.3 ± 0.412 | NS | NS |
| *sek-1* (simulated microgravity) | 11.93 ± 0.205 | *P* < 0.01 | *P* < 0.01 |
|  |  |  |  |
| wild-type (Control/NGM plates) | 13.94 ± 0.542 |  |  |
| wild-type (liquid S medium) | 14.01 ± 0.375 | NS |  |
| wild-type (simulated microgravity) | 13.98 ± 0.25 | NS |  |
| *nsy-1* (Control/NGM plates) | 13.87 ± 0.461 |  | NS |
| *nsy-1* (liquid S medium) | 14.023 ± 0.438 | NS | NS |
| *nsy-1* (simulated microgravity) | 11.85 ± 0.312 | *P* < 0.01 | *P* < 0.01 |

Note:

-NS: no significant difference

-Fifty nematodes were examined per treatment, and three replicates were performed.

**Table S2. Tissue-specific activity of PMK-1 in regulating lifespan in simulated microgravity treated nematodes**

|  | Mean lifespan (day) | Significance (*vs.* wild-type) | Significance (*vs.* *pmk-1*) |
| --- | --- | --- | --- |
| wild-type (simulated microgravity) | 14.17 ± 0.39 |  |  |
| *pmk-1* (simulated microgravity) | 10.64 ± 0.59 | *P* < 0.01 |  |
| *pmk-1Ex(*P*ges-1-pmk-1)* (simulated microgravity) | 14.04 ± 0.36 | NS | *P* < 0.01 |
| *pmk-1 Ex(*P*unc-14-pmk-1)* (simulated microgravity) | 10.99 ± 0.41 | *P* < 0.01 | NS |

Note:

-NS: no significant difference

-Fifty nematodes were examined per treatment, and three replicates were performed.

**Table S3. Effect of intestine-specific RNAi knockdown of genes encoding p38 MAPK signaling pathway on the lifespan in simulated microgravity treated nematodes**

|  | Mean lifespan (day) | Significance (*vs.* Control/NGM plates) | Significance (*vs.* VP303) |
| --- | --- | --- | --- |
| VP303 (Control/NGM plates) | 13.1 ± 0.622 |  |  |
| VP303 (liquid S medium) | 13 ± 0.354 | NS |  |
| VP303 (simulated microgravity) | 13.079 ± 0.618 | NS |  |
| *pmk-1(RNAi)* (Control/NGM plates) | 13.1 ± 0.661 |  | NS |
| *pmk-1(RNAi)* (liquid S medium) | 13.09 ± 0.483 | NS | NS |
| *pmk-1(RNAi)* (simulated microgravity) | 11.17 ± 0.332 | *P* < 0.01 | *P* < 0.01 |
|  |  |  |  |
| VP303 (Control/NGM plates) | 13.17 ± 0.602 |  |  |
| VP303 (liquid S medium) | 13.15 ± 0.325 | NS |  |
| VP303 (simulated microgravity) | 13.08 ± 0.416 | NS |  |
| *sek-1(RNAi)* (Control/NGM plates) | 13.02 ± 0.263 |  | NS |
| *sek-1(RNAi)* (liquid S medium) | 13.18 ± 0.712 | NS | NS |
| *sek-1(RNAi)* (simulated microgravity) | 10.42 ± 0.463 | *P* < 0.01 | *P* < 0.01 |
|  |  |  |  |
| VP303 (Control/NGM plates) | 13.67 ± 0.322 |  |  |
| VP303 (liquid S medium) | 13.09 ± 0.553 | NS |  |
| VP303 (simulated microgravity) | 13.05 ± 0.318 | NS |  |
| *nsy-1(RNAi)* (Control/NGM plates) | 13.275 ± 0.336 |  | NS |
| *nsy-1(RNAi)* (liquid S medium) | 13.12 ± 0.481 | NS | NS |
| *nsy-1(RNAi)* (simulated microgravity) | 11.06 ± 0.456 | *P* < 0.01 | *P* < 0.01 |

Note:

-NS: no significant difference

-Fifty nematodes were examined per treatment, and three replicates were performed.

**Table S4. Genetic interaction between PMK-1 and SKN-1 or ATF-7 in regulating the lifespan in simulated microgravity treated nematodes**

|  | Mean lifespan (day) | Significance (*vs.* Control/NGM plates) | Significance (*vs.* VP303) |
| --- | --- | --- | --- |
| wild-type (Control/NGM plates) | 14.71 ± 0.281 |  |  |
| wild-type (liquid S medium) | 14.08 ± 0.587 | NS |  |
| wild-type (simulated microgravity) | 14.67 ± 0.372 | NS |  |
| *Is(*P*ges-1-pmk-1)* (Control/NGM plates) | 14.65 ± 0.145 |  | NS |
| *Is(*P*ges-1-pmk-1)* (liquid S medium) | 14.79 ± 0.224 | NS | NS |
| *Is(*P*ges-1-pmk-1)* (simulated microgravity) | 14.18 ± 0.453 | NS | NS |
|  | Mean lifespan (day) | Significance (*vs.* Control/NGM plates) | Significance (*vs.* wild-type) |
| wild-type (Control/NGM plates) | 14.41 ± 0.432 |  |  |
| wild-type (liquid S medium) | 14.25 ± 0.367 | NS |  |
| wild-type (simulated microgravity) | 14.13 ± 0.344 | NS |  |
| *atf-7* (Control/NGM plates) | 14.37 ± 0.416 |  | NS |
| *atf-7* (liquid S medium) | 14.14 ± 0.311 | NS | NS |
| *atf-7* (simulated microgravity) | 11.92 ± 0.487 | *P* < 0.01 | *P* < 0.01 |
| *skn-1* (Control/NGM plates) | 11.99 ± 0.298 |  | *P* < 0.01 |
| *skn-1* (liquid S medium) | 11.89 ± 0.361 | NS | *P* < 0.01 |
| *skn-1* (simulated microgravity) | 7.69 ± 0.612 | *P* < 0.01 | *P* < 0.01 |
|  | Mean lifespan (day) | Significance (*vs.* wild-type) | Significance (*vs.* *Is(*P*ges-1-pmk-1)*) |
| wild-type (simulated microgravity) | 13.97 ± 0.394 |  |  |
| *Is(*P*ges-1-pmk-1)* (simulated microgravity) | 13.95 ± 0.311 | NS |  |
| *skn-1* (simulated microgravity) | 8.17 ± 0.387 | *P* < 0.01 |  |
| *atf-7* (simulated microgravity) | 11.12 ± 0.122 | *P* < 0.01 |  |
| *skn-1Is(*P*ges-1-pmk-1)*  (simulated microgravity) | 8.19 ± 0.403 | *P* < 0.01 | *P* < 0.01 |
| *atf-7;(*P*ges-1-pmk-1)*  (simulated microgravity) | 11.21 ± 0.304 | *P* < 0.01 | *P* < 0.01 |

Note:

-NS: no significant difference

-Fifty nematodes were examined per treatment, and three replicates were performed.

**Table S5. Effect of intestine-specific RNAi knockdown of *skn-1* or *atf-7* on the lifespan in simulated microgravity treated nematodes**

|  | Mean lifespan (day) | Significance (*vs.* Control/NGM plates) | Significance (*vs.* VP303) |
| --- | --- | --- | --- |
| VP303 (Control/NGM plates) | 14.16 ± 0.342 |  |  |
| VP303 (liquid S medium) | 14.08 ± 0.314 | NS |  |
| VP303 (simulated microgravity) | 14.02 ± 0.414 | NS |  |
| *atf-7(RNAi)* (Control/NGM plates) | 14.13 ± 0.405 |  | NS |
| *atf-7(RNAi)* (liquid S medium) | 13.97 ± 0.517 | NS | NS |
| *atf-7(RNAi)* (simulated microgravity) | 10.11 ± 0.287 | *P* < 0.01 | *P* < 0.01 |
| *skn-1(RNAi)* (Control/NGM plates) | 11.15 ± 0.412 |  | *P* < 0.01 |
| *skn-1(RNAi)* (liquid S medium) | 10.92 ± 0.344 | NS | *P* < 0.01 |
| *skn-1(RNAi)* (simulated microgravity) | 6.97 ± 0.405 | *P* < 0.01 | *P* < 0.01 |

Note:

-NS: no significant difference

-Fifty nematodes were examined per treatment, and three replicates were performed.

**Table S6. Primers used for quantitative real-time PCR of genes**

| Gene | Forward Primer (5’-3’) | Reverse Primer (5’-3’) |
| --- | --- | --- |
| *tba-1* | TCAACACTGCCATCGCCGCC | TCCAAGCGAGACCAGGCTTCAG |
| *act-1* | ACGACGAGTCCGGCCCATCC | GAAAGCTGGTGGTGACGATGGTT |
| *pmp-3* | TGGCCGGATGATGGTGTCGC | ACGAACAATGCCAAAGGCCAGC |
| *pmk-1* | CGACTCCACGAGAAGGAT | ATATGTACGACGGGCATG |
| *sek-1* | TGCTCAACGAGCTAGACG | ATGTTCGACGGTTTCACG |
| *nsy-1* | TGCGATGAACTACTACGG | CACCCAAATGACCAAATA |
| *atf-7* | CTGGAGAACTTGACGTGGCA | ATCCGACATTGTTCCGGCAT |
| *skn-1* | AGGCTCAACCTCAGAACATG | TACGAGTAGGCGGTCATTTC |
| *mev-1* | GGAATTCGCTTCTTAGGAT | GCAGTCTTGTTGCTCTTGT |
| *gas-1* | CTTGGTCTTTGGCTGTTGA | CTTGGTCTTTGGCTGTTGA |
| *isp-1* | GCAGAAAGATGAATGGTCC | CAGAAGCGTCGTAGTGAGA |
| *clk-1* | CACATACTGCTGCTTCTCGT | TGAACCAACAGATGAACCTT |
| *sod-1* | ACGCTCGTCACGCTTTAC | TCTTCTGCCTTGTCTCCG |
| *sod-2* | GGCATCAACTGTCGCTGT | ACAAGTCCAGTTGTTGCC |
| *sod-3* | TGACATCACTATTGCGGT | GGGACCATTCCTTCCAAA |
| *sod-4* | CACCAGATGACTCGAACA | AATGAGGCAAGAGAGTCG |
| *sod-5* | ATATTGCCAATGCCGTTC | CTCTTCACCTTCGGCTTT |

**Table S7. Primers information for DNA constructions**

| Gene | Forward Primer (5’-3’) | Reverse Primer (5’-3’) |
| --- | --- | --- |
| P*unc-14* | ATATCTAGAAGCCACTCAGCCACTTCA | ATAGGATCCCATCTGAATTCAAAGATA |
| P*ges-1* | ATAAAGCTTCCATCAGTTAAAACCTGT | ATAGGATCCCATTTTGGTGGAAGAATT |
| *pmk-1* | TACCCATGGATGTTTCCACAGACAACAAT | ACTCTCGAGCTACGATTCCATTTTCTCCT |
